# Composite estimation to combine spatially overlapping environmental monitoring surveys

**DOI:** 10.1371/journal.pone.0299306

**Published:** 2024-03-22

**Authors:** Steven L. Garman, Cindy L. Yu, Yuyang Li

**Affiliations:** 1 Bureau of Land Management, National Operations Center, Denver Federal Center, Denver, Colorado, United States of America; 2 Department of Statistics, Iowa State University, Ames, Iowa, United States of America; USDA Forest Service Southern Research Station, UNITED STATES

## Abstract

Long-term environmental monitoring surveys are designed to achieve a desired precision (measured by variance) of resource conditions based on natural variability information. Over time, increases in resource variability and in data use to address issues focused on small areas with limited sample sizes require bolstering of attainable precision. It is often prohibitive to do this by increasing sampling effort. In cases with spatially overlapping monitoring surveys, composite estimation offers a statistical way to obtain a precision-weighted combination of survey estimates to provide improved population estimates (more accurate) with improved precisions (lower variances). We present a composite estimator for overlapping surveys, a summary of compositing procedures, and a case study to illustrate the procedures and benefits of composite estimation. The study uses the two terrestrial monitoring surveys administered by the Bureau of Land Management (BLM) that entirely overlap. Using 2015–18 data and 13 land-health indicators, we obtained and compared survey and composite indicator estimates of percent area meeting land-health standards for sagebrush communities in Wyoming’s Greater Sage-Grouse (*Centrocercus urophasianus*) Core and NonCore conservation areas on BLM-managed lands. We statistically assessed differences in indicator estimates between the conservation areas using composite estimates and estimates of the two surveys individually. We found composite variance to be about six to 24 units lower than 37% of the survey variances and composite estimates to differ by about six to 10 percentage points from six survey estimates. The composite improvements resulted in finding 11 indicators to statistically differ (*p* <0.05) between the conservation areas compared to only six and seven indicators for the individual surveys. Overall, we found composite estimation to be an efficient and useful option for improving environmental monitoring information where two surveys entirely overlap and suggest how this estimation method could be beneficial where environmental surveys partially overlap and in small area applications.

## Introduction

Large-scale, long-term environmental monitoring provides sound information on the status and trend of natural resources [1, 2] to inform conservation management [3]. Data collected over time increase understanding of the composition and structure of ecosystems [2] and how natural forces of change influence variation in system properties. Data also provide a basis for determining the effectiveness of management practices, identifying appropriate changes or new management strategies to achieve desired objectives [2, 3], and detecting undesirable trends in resources so timely corrective actions can be implemented [4].

Environmental monitoring efforts tend to be probability sample surveys that use a sample size expected to provide reliable information about the monitored population [4], but over time, efforts typically can benefit from using complementary information to bolster accuracy and precision of resource estimates. Higher than anticipated resource variability and expansion of data uses for smaller areas of the population with few observations [5] often motivate combining data of a surveyed population with information from other sources. In cases where another survey overlaps the surveyed population, observations may be combined to improve accuracy and precision depending on survey designs [6]. Where it is not possible or practical to combine observations, composite estimators offer an alternative method that combines the survey population estimates. Where an overlapping survey is not available but modeled estimates can be acquired, estimators also can be used to combine population estimates of both sources. The complexity of estimators varies, but a commonality is the use of a weighted combination of two estimates where weights are based on estimate precisions. The combined information provides an improved estimate (more accurate) with lower (improved) precision. Composite estimator applications have used spatially overlapping local and regional surveys to improve estimates of the smaller scale area [7] and at both scales [8], and even to composite temporally repeated observations of a survey to improve estimates of different time periods [9–11]. Composite estimators are commonly used in what is formally called small area estimation to bolster subpopulation estimates with low sample sizes and precisions by combining them with more precise synthetic (often modeled) estimates derived from a larger portion of the surveyed population [12–19] or a similar population [20]. Composite estimation with complementary data offers environmental monitoring efforts an analytical approach to improve assessments of resource conditions without costly modification of an ongoing monitoring design, such as increasing the number of sampling sites.

The two independent terrestrial monitoring programs of the Bureau of Land Management (BLM) are examples of spatially overlapping surveys that would benefit from composite estimation of population estimates. About 68% (683,545 km^2^) of the public lands administered by the BLM are primarily grass-shrub dominated rangelands across 13 western U.S. states. For context, this is about 6.8% of the total U.S. land surface. BLM manages rangelands for multiple uses and purposes, such as permitted livestock grazing, energy development, recreation, and native habitat conservation. The current Assessment Inventory and Monitoring terrestrial (AIMt) [21] and Landscape Monitoring Framework (LMF) [22] programs were operationally implemented in 2014 to provide land-health information to inform sustainable management of BLM-administered rangelands. Funding sources, reporting requirements, and survey designs of the programs differ, but both monitor the same BLM surface-managed lands (hereafter BLM-managed lands), are probability sample surveys, provide unbiased estimates, and use similar field-based methods to collect vegetation and soil observations in a sample of sites (i.e., field plots) [23]. Combining survey data for management applications ranging from individual grazing allotments (~ 1,000–8,999 km^2^) to state-wide assessments (~ 33,000–186,000 km^2^) to provide the best possible resource estimates has been identified as a need by BLM [5]. More reliable estimates also would benefit external research and conservation efforts using the publicly available monitoring data. Although the BLM surveys entirely overlap, the observations alone cannot be simply pooled to obtain population estimates. As with all probability samples, each site in a sample contributes a specific portion to the total population. This portion must be used to weight observations when estimating population statistics (e.g., mean and variance). Combining observations of two probability surveys first requires adjusting the site portions (i.e., weights) in the context of a combined sample [6]. This adjustment requires merging survey maps of all possible sampling sites, not just those selected to be in a sample. For reasons described below (Summary of survey designs section) these maps are not available for both BLM surveys. This has inhibited properly combining AIMt and LMF observations. A practical alternative is to use composite estimation to combine the population estimates.

In this paper we first present a basic two-survey composite estimator and generalized procedures for compositing population estimates of overlapping probability sample surveys. A case study using the two BLM terrestrial surveys is then presented to illustrate a practical implementation of composite estimation. The study uses 2015–18 survey observations to obtain survey and composited estimates of 13 land-health indicators of sagebrush communities in Wyoming Core and NonCore Greater Sage-Grouse (*Centrocercus urophasianus*) conservation areas [24–26] on BLM-managed lands. The Greater Sage-Grouse (hereafter sage-grouse) is a species of conservation concern in the western U.S. [27]. State-wide Core designations bound areas with large sage-grouse population sizes and have stringent stipulations limiting energy development and overall disturbance of sage-grouse populations and habitat [26]. NonCore areas are sage-grouse population areas outside of Core and have less stringent stipulations [26]. Estimates used in our study were percent area of sagebrush communities meeting land-health standards of desired condition for 13 site-scale vegetation and soil indicators. Objectives of the study were: 1) to determine the degree of improvement in composited indicator estimates and variances relative to those of the two surveys, and 2) to determine statistical differences in land-health condition between the conservation areas to assess the implications of composite improvements in hypothesis testing. Previous studies have compared Core, NonCore energy development trends, and sage-grouse use and nesting habitat to evaluate the effectiveness of the conservation strategy since its inception in 2008 [26, 28–30]. None have involved a broader land-health assessment. We used Core and NonCore areas only for demonstration purposes but the ecological results of the study contribute to the growing knowledge base of the conservation areas.

## Study area

**Fig 1:**
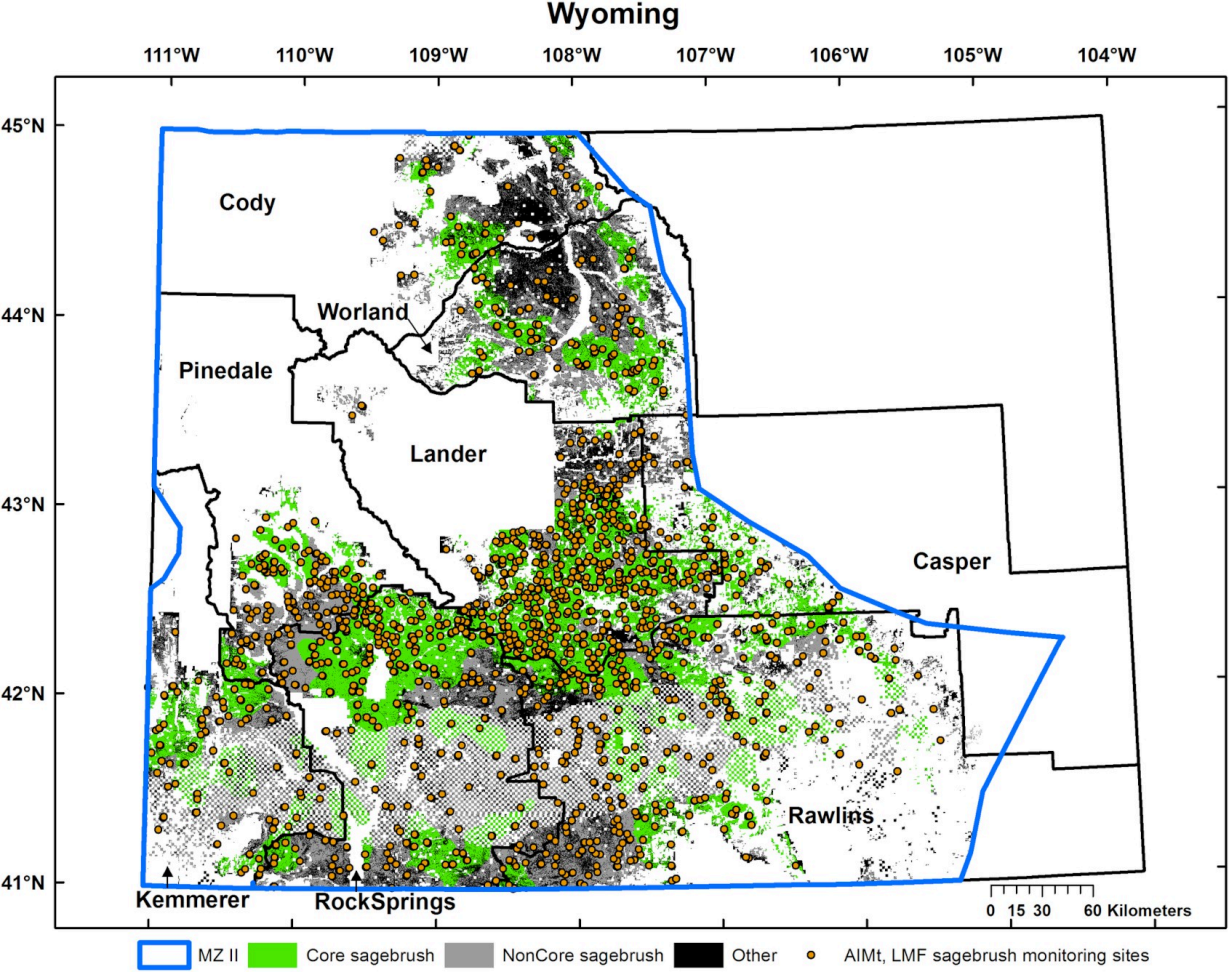
Study area. The eight Wyoming BLM field offices overlapping Western Association of Fish and Wildlife Agencies management zone (MZ) II, Core and NonCore sagebrush communities on MZ II BLM-managed lands, and the 2015–18 AIMt and LMF sample sites. The aggregate of Core and NonCore sagebrush and Other (other land-cover types) is the full extent of BLM-managed lands within MZ II. The appearance of sampling sites within Other is an artifact of map resolution.

We used the Western Association of Fish and Wildlife Agencies (WAFWA) sage-grouse management zone (MZ) II [31] in Wyoming (Fig 1) for our case study. This management zone spans the multiple basins of southwest and northcentral Wyoming and overlaps BLM-managed land in eight administrative units called field offices (Fig 1) that ranged from 3,746 to 14,593 km^2^. The Wyoming basins contain some of the most intact, contiguous expanses of sagebrush steppe remaining in the western U.S. [32]. We limited our case study to sagebrush communities because of their importance to sage-grouse and other sagebrush-obligate species [33, 34]. Also, this avoids confounding effects by including other communities with different vegetation composition and structure and that can have different land-health standards. Sagebrush communities were delineated from land-cover mapping [35] and span 26,369 km^2^ of Core (87% of BLM MZ II Core) and 20,970 km^2^ of NonCore (64% of BLM MZ II NonCore, Fig 1). Native bunchgrasses are dominant followed by Wyoming big sagebrush (*Artemisia tridentata wyomingensis*) and basin big sagebrush (*A*. *t*. *tridentata)* at lower elevations, and mixed mountain shrubs, which include mountain big sagebrush (*A*. *t*. *vaseyana*), at higher elevations [32]. Pockets of deciduous and coniferous woodlands and forests occur on the numerous escarpments across the basins [36].

## Methods

### Composite estimator

A basic composite estimator for two independent survey estimates is;

$${Ŷ}_{c}={\lambda Ŷ}_{1}+{(1-\lambda )Ŷ}_{2},$$ (1)

where ${Ŷ}_{c}$ is the composite estimate and ${Ŷ}_{1}$ and ${Ŷ}_{2}$ are independent, design-based population estimates from survey #1 and #2, respectively, of the same variable (e.g., a resource attribute such as % shrub cover). Eq (1) is a special case of an estimator that can composite any number of surveys [37]. The composite weight of the survey #1 estimate is λ and 1- λ is the composite weight of the survey #2 estimate. Hereafter we refer to these two weights collectively as composite weightings. A composite estimate can be for a population total (e.g., amount of woody biomass), population mean (e.g., mean % grass cover), or a population proportion (e.g., % of population area meeting a land-health standard). The designation of surveys as #1 and #2 is arbitrary.

Composite weightings that maximize precision (minimize variance) of a composite estimate are obtained from variance weighting when estimates are unbiased or from Mean Squared Error (MSE) weighting to correct for bias [11]. Assuming unbiased estimates, a variance weighted λ is obtained by;

$$\lambda =\frac{{\hat{\sigma }}_{{Ŷ}_{2}}^{2}}{{\hat{\sigma }}_{{Ŷ}_{1}}^{2}+{\hat{\sigma }}_{{Ŷ}_{2}}^{2}},$$ (2)

where ${\hat{\sigma }}_{{Ŷ}_{2}}$ is the standard error (SE) of the survey #2 estimate, ${\hat{\sigma }}_{{Ŷ}_{1}}$ is the SE of the survey #1 estimate, and λ is a positive fraction (0 ≤ λ ≤1). The square of SE is the estimate of the variance of an estimator, and conversely, the square root of this variance is the SE of an estimate. The sum of λ and 1- λ is always one. Eq (2) provides optimal weightings that give the more precise estimate a higher weighting.

The composite-weighted pooled variance of a composite estimate, $\hat{V}({Ŷ}_{c})$, is;

$$\hat{V}({Ŷ}_{c})=\lambda^{2}{\hat{\sigma }}_{{Ŷ}_{1}}^{2}+{\left(1-\lambda \right)}^{2}{\hat{\sigma }}_{{Ŷ}_{2}}^{2}.$$ (3)

The SE of a composite estimate is $\sqrt{\hat{V}({Ŷ}_{c})}$.

**Fig 2:**
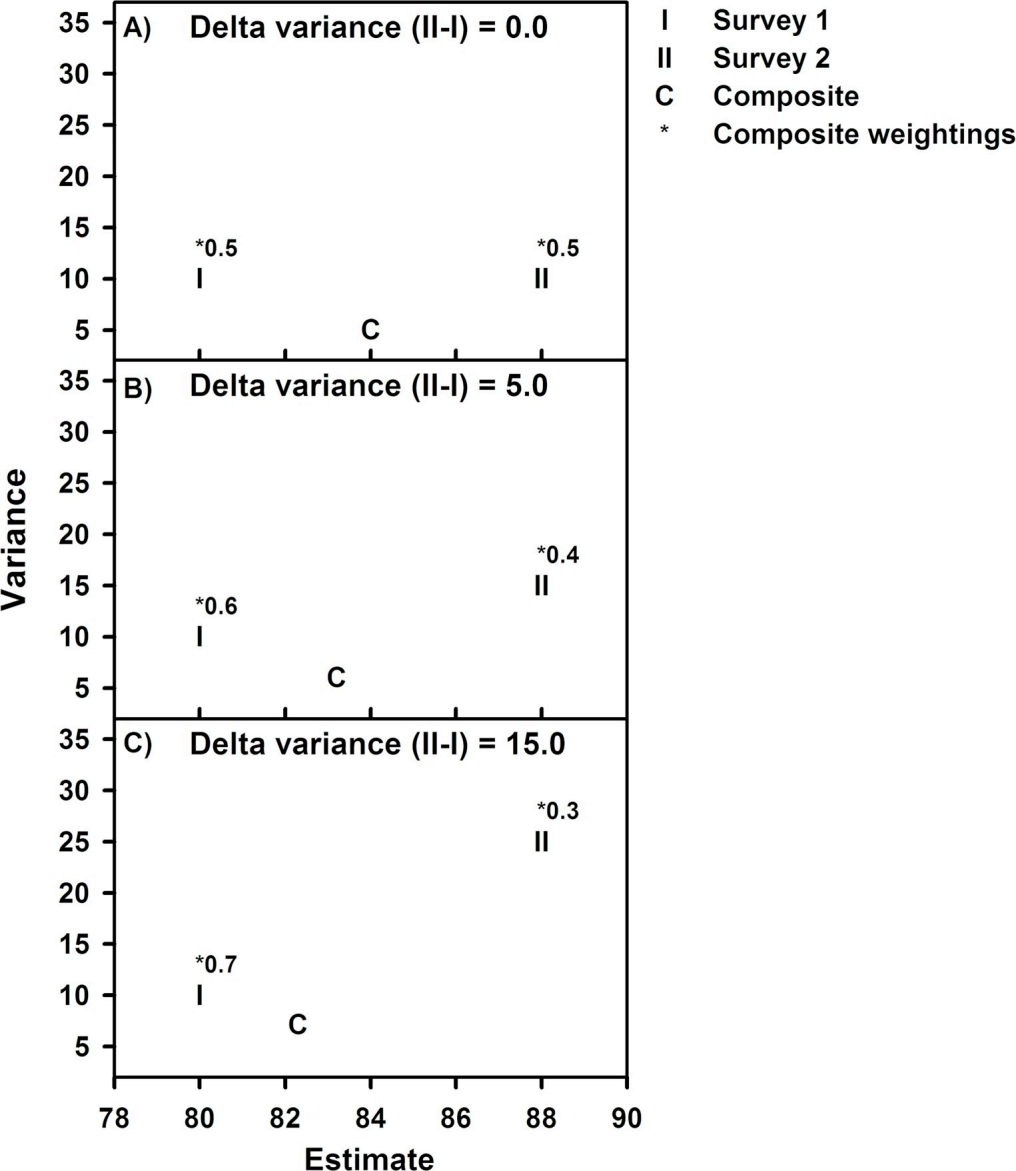
Examples of improvement in composite estimates. Illustration showing the influence of the difference between survey estimate variances on composite weightings and improvements of the composite estimate and variance.

The improvement of a composite is relative to each survey estimate. When survey variances are equal, both estimates are equally reliable and have equal weighting in deriving a composite estimate. In these cases, a composite is a balanced improvement of the surveys where composite variance is a 50% reduction relative to either survey (composite weightings = 0.5) and the composite estimate is an arithmetic average of the two surveys (Fig 2A). To generalize, as the difference between the two survey estimate variances increases, the survey estimate with a higher precision (lower variance) has an increasing composite weight (>0.5) and a composite trends toward the variance and estimate values of this survey (I in Fig 2B and 2C) and away from the values of the survey with lower precision (higher variance) because of its decreasing weighting (<0.5, II in Fig 2B and 2C). The composite variance, however, will always be less than that of either survey. The magnitude of the survey variances and their difference determines the composite weightings and how much the composite variance differs from that of either survey. The mathematical basis of composite optimal weightings and variance improvement is provided in S1 Appendix. The composite estimate is more accurate because it is based on a larger number of observations and is more heavily weighted by the more precise survey estimate. Similarly, the magnitude of survey estimates and their difference determines how much the composite estimate differs from that of either survey.

Composite estimation requires scrutiny for bias in survey estimates to determine proper weighting methods. If estimates are independent and unbiased, then variance weighting provides the best linear unbiased estimate [38]. If there is evidence of bias, it must be corrected for using MSE weighting. Bias in estimates can occur for various reasons, such as improper randomization of sample site selection and measurement error by field crews. The ultimate effect is it results in an inaccurate, skewed population estimate. The computation of MSE weighting can be complex because it involves estimating both variance and bias from the two surveys which typically is difficult [11, 15, 39]. In short, the weighting minimizes the effect of bias MSE so a relatively unbiased and accurate composite estimate can be obtained. A practical outcome of not correcting for bias is the risk of reporting a highly inaccurate composite estimate that promotes conservation actions when none are needed or that does not motivate corrective actions when in fact a resource is impaired.

We summarize the stepwise procedures to obtain composite estimates with the basic estimator (Eqs (1–3)) for overlapping surveys assuming upfront that estimates are not biased. The procedures serve as a general guide. Various nuances of surveys in an application inevitably will require additional procedures. Procedures require the sample frame, observations, and sample weights of both surveys. As background, environmental probability sample surveys use a sample frame that physically bounds the population to be monitored. A population, for instance, can be all vegetation communities or nesting habitat of a bird species. A frame is typically a digital map generated from existing resource maps or aerial imagery. The frame is used to select a randomized sample consisting of *n* number of sites. The probability of a site being selected in a sample translates to the specific portion contributed to the total population. This portion is referred to as the sample weight. Observations of variables are to be recorded at each site, but there can be instances where a site is not the targeted population (due to frame error) or is inaccessible in the field due to safety (steep slopes) or trespass reasons. These sites are unobserved. The sum of sample weights of observed sites represents the sampled population area. The sum of sample weights of unobserved sites represents the area of a population not of interest, or for inaccessible sites, the area where the population(s) is unknown. There are exceptions where remote sensing information is used to determine inaccessible targeted population sites and their sample weights are included in the population area total.

Step 1—Identify the common sampled population. The region where the two survey frames overlap identifies the common sampled population area [6]. The overlap region may be a subset of one or both survey frames. GIS clipping of original frames and samples to the overlap region is typically performed, so we refer to the common sampled population information as clipped frames and samples. If surveys sampled the same population, then all observations of the clipped samples are used to obtain estimates for composite estimation. There can be instances, however, where the targeted population within the overlap area differs between the two surveys. For instance, a survey may only sample older forest stands that could not be reliably distinguished in source maps. For practical reasons, an imperfect frame consisting of all forest stands was used to obtain a sample. Sites in younger stands are rejected in the field and not observed. An overlapping survey may have sampled all forest stand ages. In these cases, the common sampled population is older forest stands and only estimates using observations in these stands can be used in composite estimation. Including codes in the clipped sample data sets to indicate sites of the common population can ensure only these are subsequently used to obtain survey estimates. Only the clipped frames and the clipped samples and their original sample weights are carried forward in compositing procedures. The clipped samples include all observed and unobserved sites and their original sample weights which is necessary for proper sample weight adjustments in subsequent steps.

Step 2—Calibrate sample weights to the clipped frame area of the common sampled population. When the extent of a clipped survey frame is less than the original frame area, the sample weights of all sites in the clipped sample must be calibrated to the clipped frame area. This is usually done with ratio adjustments (scales sample weights to frame area), but approaches can differ to accommodate survey design differences. After any necessary calibration, the sum of the sample weights of observed sites in a clipped sample is the estimated area of the common sampled population. The areal estimates should be the same for both surveys for a composite estimate to be meaningful and useful. If this is not the case, an additional sample weight adjustment must be performed to equilibrate areal estimates of the two clipped samples. Generally, the areal estimate considered the most accurate is used to adjust the weights of the clipped sample of the other survey, but other approaches may be required for complex survey designs. In all cases, discerning reasons for the different estimates is necessary to determine an appropriate adjustment procedure.

Step 3—Determine the required number of composite weightings. If an application involves only one variable, then variances (SE^2^) of the survey estimates are required to derive composite weightings (Eq (2)). There are three basic options for generating composite weightings when more than one variable is used in an application: use separate composite weightings for each variable, use the composite weightings of the most important variable for all variables, or use the average of the weightings of multiple important variables for all variables. Using separate composite weightings provides the minimum variance for each composite estimate and requires obtaining variances for every survey estimate. The other options require obtaining fewer variance estimates which is advantageous when compositing many variables.

Step 4—Obtain survey estimates and their variances (SE^2^). Estimate and variance for the variables selected in Step 3 are obtained using software packages designed for analyzing probability samples. Using a variance estimator that provides the most precise and unbiased variance given the design of a survey is critical given the use of variance weighting in composite estimation (Eq (2)). Variance estimators can differ between surveys. Examples of variance estimators for probability sample surveys include the local mean variance estimator [40] that produces a more precise estimate for spatially-balanced samples (i.e., no clumping of sites), the simple random sample estimator for a sample that is not well-balanced (i.e., has some clumping), and replicate variance estimators (e.g., [41]) for more complex surveys that use multiple calibrations to obtain sample weights.

Step 5—Obtain composite estimates for each variable. Composite weightings are obtained using the survey variances (Eq (2)), and along with survey estimates, are used to acquire composite estimates (Eq (1)) and their precision (Eq (3)).

### Case study

#### Summary of survey designs

The AIMt and LMF surveys were designed independently, have different goals, and different designs. The primary goal of the AIMt program is to assess the effectiveness of BLM field office resource management plans [42–44]. The AIMt program uses similar but separate surveys for each BLM field office to accommodate management’s needs. Because surveys do not physically overlap, observations and sample weights can be aggregated to make inferences at large spatial extents. Surveys generally use a stratified sample frame that bounds all upland vegetation communities on BLM-managed lands (e.g., rangelands, woodlands, semi- to closed-canopy forests). The AIMt surveys are one-stage designs, where a spatially-balanced [45] sample is selected from the stratified frame. The LMF program uses state-specific sample frames, but otherwise monitoring designs are consistent. The goal of LMF is to report on the status of rangeland indicators at three reporting units: state, ecoregion, and sage-grouse management zones [22]. An LMF state frame bounds all BLM-managed lands in a state that is the same area as the combined sample frames of AIMt field office surveys in a state. The frame is stratified by prime sage-grouse habitat and other. A state survey is a two-stage cluster design that first selects ~65-ha rectangular areas, termed segments, then locates three sites in each segment labeled one to three to designate observation order. Up to two sites are to be observed. The third site is observed only if one or both of the first two sites were non-rangeland or inaccessible and not observed. Spatially-balanced procedures are used for both stages [22].

In addition to disparate strata and site selection methods, surveys have different target populations and methods to estimate rangeland area. First, LMF only monitors rangelands, which it defines as grass-shrub dominant areas with low (<40%) tree overstory [46]. Non-rangeland sites are determined in the field and not observed. The community type of AIMt sites is not explicitly recorded; however, LMF’s criteria can be applied to the recorded observations to determine compatible rangeland sites. Second, LMF field crews visually score rangeland status of all segment sites, even inaccessible sites if they can be seen from a distance or from aerial imagery. Rangeland area is the weight sum of these sites and is used to obtain the final weights of observed sites (S2 Appendix). The AIMt program lacks a similar scoring requirement. Inaccessible sites are treated as unknown population area. Rangeland area is the weight sum of only observed sites. In overlap areas with inaccessible sites and some are rangeland, LMF’s estimate of rangeland area will be higher and more accurate than AIMt’s estimate.

Combining observations of the two surveys would require first merging the two sample frames and then re-calculating sample weights for each unique spatial combination of AIMt and LMF strata and all possible LMF first-stage segments [6]. LMF state frames, however, only consist of the segments for the initial 10-year design (2014–23). Initial clipping of a grid to the BLM-managed land parcels, which are highly irregular in shape and size, resulted in segments of variable shapes and sizes. For practical reasons, segments for the initial design were randomly selected from the clipped grid then manually edited in GIS for geometric consistency [22]. Cost and time constraints have precluded producing wall-to-wall segment mapping for the 13 LMF state frames. Combining survey population estimates instead of observations is thus more practical and cost-effective.

#### Data sources and preparation

All case-study data were acquired from the monitoring and GIS archives at the BLM National Operations Center, Denver, CO between 8/2018 and 12/2020 and are in the public domain. The AIMt survey used in this study was the aggregate of eight BLM field office surveys (Fig 1). Observations, site location, and pre-calculated indicators of the surveys are available from the BLM Geospatial Business Platform Hub AIM Page website (https://gbp-blm-egis.hub.arcgis.com/pages/aim). A web portal for the raw field data, design information, and related spatial data (e.g., survey frames, sage-grouse conservation areas, WAFWA management zones) has not been completed but data are available upon request from the Terrestrial AIM Lead (contact information available at: https://www.blm.gov/aim/strategy).

We used 10 vegetation and soil indicators of land-health of sagebrush steppe [47, 48] and two shrub indicators of nesting habitat of sagebrush-obligate songbirds [33], which similar to sage-grouse are species of conservation concern (Table 1). Habitat attributes of species are considered biotic integrity indicators of land health. We also used an annual forb-grass cover indicator to be inclusive of non-tree life forms not included in the other indicators although it is not commonly used by BLM (Table 1). Twelve of the 13 indicators were pre-calculated or generated by combining pre-calculated indicators. Values of the mean shrub height indicator were calculated from the AIMt and LMF raw field data. We obtained benchmarks that specify the indicator values ascribed to desired conditions from a synthesis of Wyoming land-health standards [48] and previous resource condition reports [47], and from the literature for songbird indicators [33]. We used an arbitrary benchmark of ≥5% for annual forb-grass cover. Applying benchmarks to indicator observations scored each as meeting and not meeting land-health standards. The sample weights of meeting observations and the areal extent of sagebrush in a conservation area provides the estimate of the percent area meeting standards for each indicator. These were the estimates used in the case study.

**Table 1:** Site-scale indicators of land health.

Indicator (units)	Description	Benchmark
Perennial grass cover (%)	Any hit of perennial grass species	≥10
Perennial grass height (cm)	Mean height of perennial grass species	≥18
Sagebrush cover (%)	Any hit of *Artemisia* spp.	5–25^1^
Sagebrush shrub height (cm)	Mean height of *Artemisia* spp. shrubs	10–80^1^
Perennial forb cover (%)	Any hit of perennial forb species	≥5
Shrub cover (%)	Any hit of shrub species	≥20
Shrub height (cm)	Mean height of shrub species	≥35
Herbaceous height (cm)	Mean height of herbaceous plant species	≥18
Large foliar gaps cover (%)	Cover of foliar canopy gaps > 2 m	≤10
Total foliar cover (%)	First hit of any vascular plant species	≥40
Bare ground cover (%)	First hit of soil surface (no obstruction by live or dead vascular or non-vascular plant species or rock)	≤50
Soil aggregate stability (index)	Average soil stability of Slake-test subsamples [23]; index from 1 (low stability) to 6 (high stability)	≥4
Annual forb-grass cover (%)	Any hit of annual forb or annual grass species	≥5

Indicators used in composite estimation and compared between Wyoming Core and NonCore sagebrush communities on BLM-managed lands in WAFWA MZ II. Vegetation and ground cover were sampled using the line-point intercept method which uses multiple pin drops along transects. Under description, “First hit” is the first pin intercept (uppermost vegetation layer), and “Any hit” is any pin intercept from the upper layer to the ground. Canopy gaps, plant heights, and soil stability were measured along transects. Benchmark is a minimum, maximum, or range of values ascribed to desired condition of the indicator.

^1^ ranges are inclusive.

Our compositing procedures mirrored the five general steps outlined above.

Step 1. Because the extent of AIMt and LMF frames were identical we only had to clip frames and samples to sagebrush areas in the two conservation areas. This produced AIMt clipped frames and samples for Core and NonCore, and the same for LMF clipped frames and samples. After scoring the rangeland status of AIMt sites using LMF’s rangeland criteria the total number of observed rangeland sites in sagebrush was 1000 in Core (539 AIMt, 461 LMF) and 624 in NonCore (249 AIMt, 375 LMF).

Step 2. We re-calculated the LMF sample weights of clipped samples using LMF’s state-level weighting procedures with minor modifications to expedite calculations to obtain only state-level weights (S2 Appendix). The estimates of rangeland population area within the clipped frames were 26,224 km^2^ in Core and 20,467 km^2^ in NonCore. AIMt sample weights were calibrated to strata area of a clipped sample frame. The AIMt population estimates of rangeland were two and four percent lower in Core and NonCore, respectively, than LMF estimates. Because the AIMt under coverage largely stemmed from inaccessible sites, we used the more accurate LMF rangeland area estimate to equilibrate the weight sum of observed AIMt and LMF rangeland sites in a conservation area (S3 Appendix).

Steps 3–4. We used separate composite weightings (Eqs (1,2)) for each indicator composite estimate to maximize precision. For the clipped AIMt samples, indicator estimates of percent area meeting standards and variances were obtained using the simple random sample variance estimator of the *cat_analysis()* function in the R contributed package *spsurvey* [49, 50] because aggregating the eight BLM field office surveys resulting in some clumping in the clipped samples. We used LMF’s delete-a-group jackknife replicate variance estimation method [22, 41] to obtain indicator estimates and variances. The replicate method uses subsets of a sample to estimate variance. Sample weights of these subsets require adjustments to match the population area of the clipped sample. Procedures for this adjustment are presented in S2 Appendix.

Step 5. Using the 52 survey estimates and SEs (13 indicators X two surveys X two conservation areas), composite estimates and their variances were obtained (Eqs (1–3)) for the 13 Core indicators and the 13 NonCore indicators in sagebrush communities.

### Data analysis

We summarized survey indicator estimates and their variances separately for Core and NonCore and composite estimation improvements across the 26 composited estimates. By survey and conservation area, we obtained the average and range of indicator estimates of percent area meeting standards and their variances. By conservation area, we calculated survey differences as the absolute difference between each of the 13 paired indicator estimates and their variances and tallied means and ranges of differences. Composite improvements were assessed separately relative to the more and the less precise survey estimates of an indicator. For each composite estimate, improvements were determined as the absolute difference between the composite and a survey estimate and between a survey estimate variance and composite estimate variance which is always lower. Aggregating across all indicators and the conservation areas, we summarized improvements using biplots to show the magnitude of composite variance improvement with increasing differences between survey variances and the magnitude of composite estimate improvement with increasing differences between survey estimates.

Significant difference (*p* <0.05) between Core and NonCore for each of the 13 composited indicator estimates was determined with the Z-Score statistic (2-tailed tests). We performed the same comparison using only the AIMt survey estimates and only the LMF survey estimates to assess how statistical outcomes using composite estimates differed from using the surveys individually. We developed an R program (S1 Code) to derive all survey and composite estimates and SEs and to perform all Z-Score tests using the AIMt (S1 Table) and LMF (S2 Table) data sets. All estimates, SEs, and test results are reported in S3 Table.

## Results

There were distinctive differences between the surveys in each conservation area. In Core, the AIMt survey had 78 more observations, lower estimate variance, and a higher number of more precise estimates compared to the LMF survey (Table 2). In NonCore, LMF had 126 more observations, much lower estimate variance, and all but one estimate was more precise compared to the AIMt survey (Table 2). On average, differences between survey estimates and variances were two to three times higher in NonCore than in Core (Table 2).

**Table 2:** Survey summary statistics for Core and NonCore indicator estimates in sagebrush communities.

Conservation area/Survey	Total no. of observations	Estimate of percent area meeting standards	Variance	No. more precise estimates	Absolute survey differences	
					Percent area meeting standards	Variance
Core						
AIMt	539	70.4 (20.0–98.0)	4.0 (0.7–6.8)	8	3.1 (0.5–12.0)	4.0 (0.3–10.1)
LMF	461	69.0 (24.7–96.6)	7.4 (0.7–15.8)	5		
NonCore						
AIMt	249	64.4 (32.0–93.3)	21.6 (5.2–27.7)	1	5.9 (0.3–13.4)	12.5 (1.9–23.8)
LMF	375	62.8 (21.9–91.8)	9.3 (3.4–17.2)	12		

The average and range of the 13 survey indicator estimates of percent area of sagebrush meeting land-health standards and their variances are shown by conservation area. Absolute survey differences are the average and range of the absolute difference in the 13 indicator estimates and variances between the two surveys within the same conservation area. Total no. of observations are sample sizes. The number of indicator observations scored as meeting standards, however, varied among surveys and conservation areas (S3 Table).

**Fig 3:**
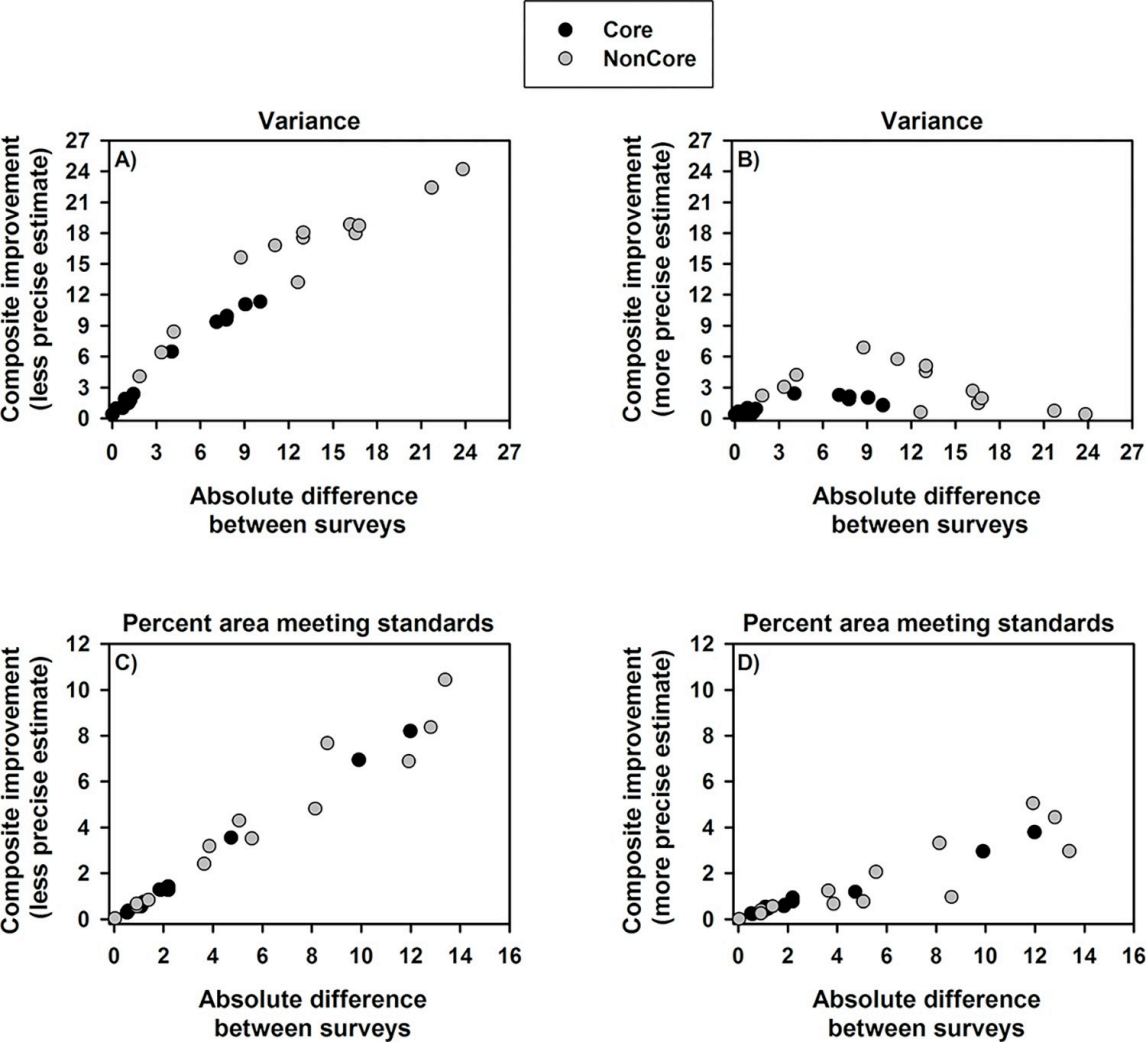
Composite improvement in variance and estimate. Composite improvements relative to the 13 AIMt and 13 LMF indicator estimates and variances by conservation area. Composite improvement in variance (graphs A-B) was the difference between survey variance and composite variance (composite variance is always less than survey variances). Composite improvement in an estimate was the absolute difference between the composite and a survey estimate (graphs C-D). Improvements relative to the less precise survey estimates are on the left and relative to the more precise survey estimates are on the right.

Composite improvements varied among indicators and the conservation areas. Composite improvement in variance relative to less precise survey estimates ranged from 0.3 to 24 units (Fig 3A) with more than a six-unit improvement for 18 composite estimates (Fig 3A). Most of these were relative to NonCore AIMt estimates which were predominantly less precise, but also to six LMF estimates which were less precise mostly in Core (Fig 3A). Composite improvement in variance relative to the more precise survey estimates was much less, with only one estimate (AIMt in NonCore) with more than a six-unit difference (Fig 3B). Improvement in composite estimates relative to the less precise survey estimates was up to 10 percentage points but only by less than two points for 14 composite estimates (Fig 3C). The larger improvements of more than six percentage points were relative to four NonCore AIMt indicator estimates and two Core LMF indicator estimates (Fig 3C), all of which had a nine to 14 percentage points difference between paired survey estimates. Composite estimate improvements relative to the more precise survey estimates were smaller with less than two percentage points improvement for 19 estimates (Fig 3D). The biplots illustrate not only larger but also a more linear trend in composite improvements over the less precise (Fig 3A and 3C) compared to the more precise estimates (Fig 3B and 3D) as differences between survey variances and estimates increase (survey variance and estimate differences tended to be correlated). This reflects the different potentials for improvement as composite estimates and variances trend away from the less and toward the more precise survey estimates.

**Fig 4:**
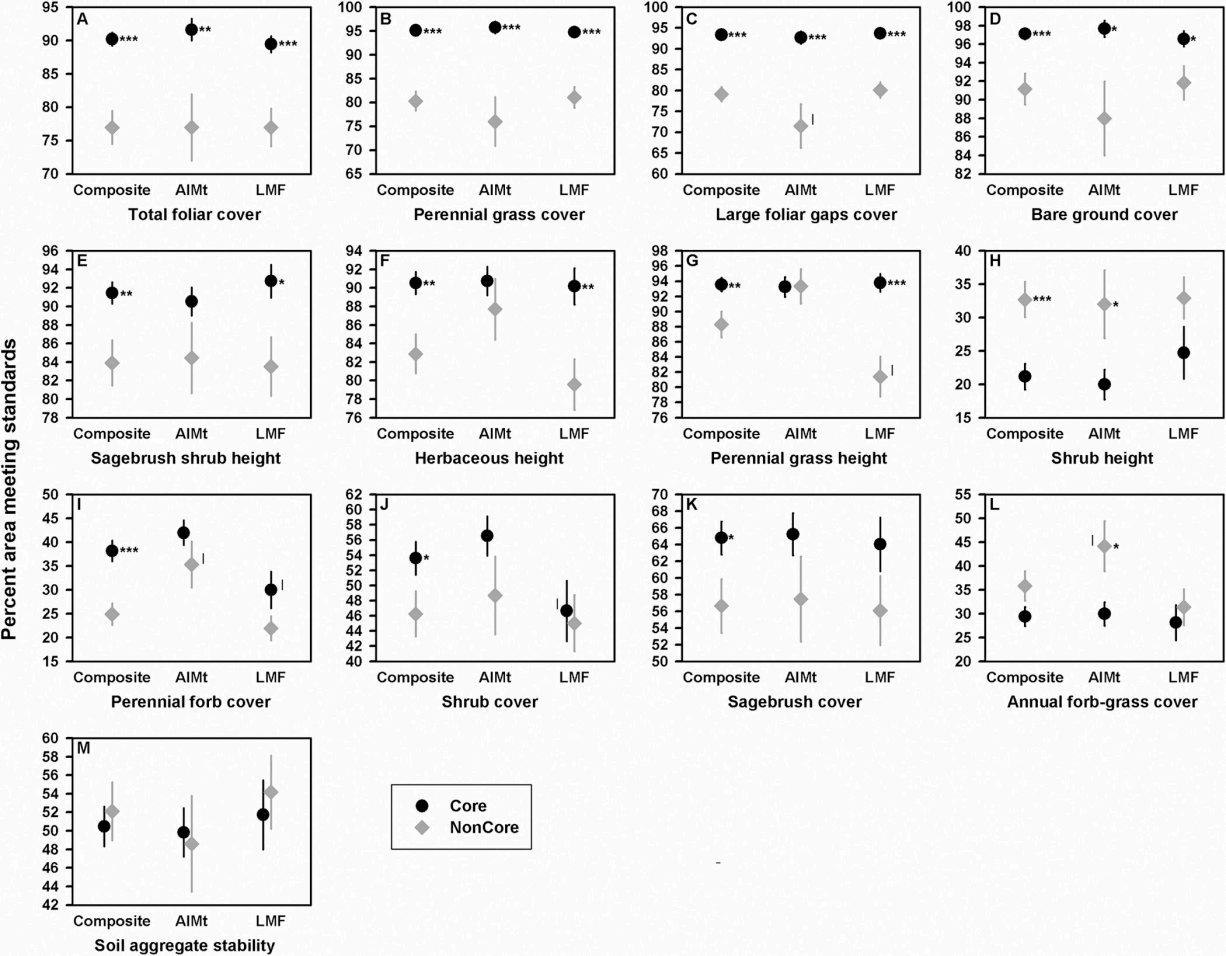
Comparisons of indicator estimates of percent area meeting standards between Core and NonCore sagebrush communities. Results of indicator comparisons using composite estimates and estimates of the individual surveys. The error bar is ± 1 SE. The Z-Score statistic determined statistical difference in means (2-tailed test); * indicates *p* <0.05; **, *p* <0.01; ***, *p* <0.001. The six survey estimates that differed from the composite estimate by more than six percentage points are noted by an I next to the survey estimate. All estimates, Z-Score statistics, and *p*-values are presented in S3 Table.

We found 11 of the 13 indicator estimates to be significantly different (*p* <0.05) between Core and NonCore sagebrush communities when using composite estimates (Fig 4). Core had higher estimates for 10 indicators. Only the shrub height indicator had a higher estimate in NonCore (Fig 4H). Comparisons using the survey estimates only found six (AIMt) and seven (LMF) indicators to be significantly different (*p* <0.05). Four indicators were significantly different between the conservation areas in all three comparisons (Fig 4A–4D) and four indicators were significantly different using the composite estimates and one of the surveys (Fig 4E–4H). Across these eight indicators, precision improvement of a composite was readily apparent for all NonCore survey estimates and even for some of the Core estimates (Fig 4A–4H). Two of the six larger improvements in estimates occurred in this group of indicators and are identified in Fig 4 by an I next to the survey estimate (Fig 4C and 4G). There were three indicators with significant differences using composite estimates but no differences using either survey (Fig 4I–4K). For these indicators, composite improvements especially over three survey estimates (Fig 4I and 4J) provided evidence in support of significant differences. For the annual forb-grass cover indicator, only the AIMt survey found a significant difference (Fig 4L). The composite estimate of this indicator was a large improvement (about 10 percentage points less) over the AIMt NonCore estimate, and this resulted in similar Core, NonCore composite estimates (Fig 4L). There were no differences in the soil aggregate stability indicator in all three comparisons (Fig 4M).

## Discussion

Results of this study illustrate how composite estimation can be used to provide more accurate and reliable indicator estimates and the implications of these improvements in hypothesis testing. All composite estimates had improved variances which is expected because they were based on the combined sample sizes of the two surveys. However, we only found large improvements (decreases of six to 24 units) relative to 37% of survey estimate variances (19 of 52). Most of these improvements were over NonCore AIMt estimates that tended to be the least precise because of the small sample size (249) in the clipped sample. All composite estimates were also improvements. The magnitude of improvements, however, were small with only six (three percent) composite estimates with more than a six percentage point difference from the less precise survey estimate. Most of these were AIMt in NonCore which differed from LMF estimates by nine to 14 percentage points. We expected most survey estimates to be about similar within a conservation area because monitoring programs use the same data collection protocols and surveys use a spatially-balanced sample that collectively minimizes the chances of large discrepancies due to crew and sampling bias. Overall, improvements in composite estimates and variances resulted in finding land-health conditions of 10 indicators to differ between the conservation areas, whereas the individual surveys found fewer differences. From an applied perspective, reporting the results of just one of the surveys would incorrectly lead management to conclude that conditions for about half of the indicators were similar between the sagebrush communities of the conservation areas and also notably provide misleading estimates of the percent area meeting standards for three indicators (e.g., AIMt—Fig 4C, 4I and 4L; LMF—Fig 4G, 4I and 4J). If a management objective was to use the conditions of Core, which supports larger sage-grouse populations, as an approximate target for NonCore, the reported results may not trigger management concerns because of the proportion of indicators in similar condition. Results with composite estimates show that conditions of most indicators are in fact different. If objectives were specific to key indicators, such as maintaining large foliar gaps cover in desired conditions for at least 80% of sagebrush communities, results of the AIMt survey in NonCore may unnecessarily trigger management concerns (Fig 4C). This would not occur using the more accurate composite estimate (Fig 4C). Reporting the results of both surveys would provide conflicting conclusions about indicator conditions and differences between the conservation areas and would be difficult for management to interpret. Compared to the individual surveys, composite estimation in our case study provided a more precise understanding of land-health conditions of sagebrush communities in the conservation areas and of area differences.

Using composite estimation does come with an operational cost in terms of additional data processing compared to using just one survey and may not always provide a substantial improvement in all resource estimates. The total foliar and perennial grass cover indicators in our study are examples where the composite and both survey estimates in a conservation area were about the same with only slightly different variances (Fig 4A and 4B). In large-scale, long-term environmental monitoring efforts, compositing all available information is still advantageous even if composite improvements are small for some or most resource indicators. Over the long term, resource estimates based on all available information provide a more accurate and reliable foundation for making conclusive statements about resource conditions and for detecting trends in conditions. The improved precision from combining complementary data especially increases the statistical power to detect small trends [51] over a shorter time period [52] which is paramount to the conservation of natural resources. In practice, a significant but small undesirable trend can trigger an investigation of the underlying reasons and the imminent risk to a resource [53]. Investigative efforts may consist of a more comprehensive analysis of potential drivers and stressors known or suspected to influence the resource to better understand the short- and long-term implications. Where possible, efforts may entail targeted monitoring of the resource to determine if the trend is largely confined to specific areas or is widespread to gauge the risk to the larger population. Small trends deemed to pose a near-term and costly risk can trigger immediate implementation of preventive management actions to reduce the chances of a resource becoming irreversibly compromised [53]. Overall, the cost of losing resources and of restorative management actions for large-scale ecosystems is substantially greater than the time and effort required to enhance ongoing monitoring efforts with additional resource information.

Our case study was an example of completely overlapping surveys, but composite estimation can be beneficial when only portions of surveys overlap. Composite estimates within the overlap region would be an improvement over individual survey estimates. For each survey, adding the improved estimates to those obtained for outside the overlap area could enhance summary statistics reported for the full extent of survey frames. An example of partly overlapping sample surveys is the three wadeable stream monitoring efforts in northwestern USA. The U.S. Forest Service (USFS) Interagency PACFISH/INFISH Biological Opinion Effectiveness Monitoring Program (PIBO) monitors multiple Federal ownerships in the interior Columbia River and upper Missouri River basins [54, 55]. The USFS Aquatic and Riparian Effectiveness Monitoring Program (AREMP) monitors multiple ownerships within the Northwest Forest Plan area, which includes west-side Oregon and Washington, and northwestern California [55, 56]. The BLM’s AIM Lotic (AIMl) survey only monitors streams on BLM-managed lands [57]. The AIMl sample frame partly overlaps the AREMP frame in western Oregon and the PIBO frame in eastern Oregon and parts of Idaho. The frame overlap areas are about 10,000–18,000 km^2^ with 100–200 sites in each survey (estimated from Scully et al. [58]). There is an ongoing effort to integrate data from these surveys to facilitate regional aquatic assessments [59]. Where combining observations of AIMl and the other surveys is not feasible because of design differences, composite estimation of population estimates offers a way to bolster estimate precision in overlap areas on BLM-managed lands. Adding these estimates to survey estimates outside the overlap additionally could enhance frame-level statistics of all three surveys.

Another beneficial use of composite estimation is in small area estimation (SAE), where the focus is on improving estimates of a population subset with a limited number of observations using information from the larger population. There are many SAE techniques but a common one is to use a composite estimator to combine low-precision direct survey estimates of a subset with more precise modeled synthetic estimates. Synthetic estimates are acquired by applying statistical relationships developed between auxiliary variables (e.g., biophysical attributes, airborne and satellite data products) and observations in the subset and in some portion or all of the remaining population. The estimator balances the unbiasedness of direct and the precision of synthetic estimates to provide improved estimates for the subset [19]. The forest inventory community over the past 20 years has researched SAE techniques to acquire more reliable estimates of forest attributes at smaller spatial scales (county level, national forests, forest-stand level) than those provided by national forest inventories, such as the USDA Forest Inventory and Analysis (FIA) program, and by state forest inventories [17–19]. The nationwide sampling intensity of one FIA plot per 2,438 ha on multiple ownerships provides insufficient data at within-state scales for reliable estimates but at the same time provides a large regional sample for SAE applications. Synthetic estimate applications using several compositing techniques (similar to Eqs (1–3)) have demonstrated the utility of SAE with FIA data for improving county and state estimates of forest attributes [17, 18]. Other environmental monitoring efforts possibly could take advantage of these techniques either directly or as a template for customizing SAE applications, such as the National Park Service Inventory and Monitoring Vital Signs program (NPS I&M) [2]. The limited number of FIA plots in most forested National Parks outside of Alaska precluded using these data for the forest vegetation vital sign. Parks either lack plot-based forest monitoring due to funding constraints or were able to implement their own survey (e.g., [60]) at least on a limited basis. Where the number of FIA plots is sufficient for reasonable direct estimates for a range of forest communities in a National Park, applying FIA SAE compositing techniques could provide forest condition information where monitoring is lacking or expand on the information currently provided by monitoring a few focal communities.

Composite estimation may also be beneficial when synthetic estimates can only be obtained from non-overlapping complementary surveys. An example is the potential to increase the precision of survey estimates of rangeland indicators in western National Parks using AIMt and LMF data to obtain synthetic estimates. The NPS I&M program monitors and reports on select ecological sites (ecosites) in 17 of the Colorado Plateau National Parks (e.g., Grand Canyon, Canyonlands) using 20–60 sites per ecosite [61, 62]. A recent assessment has shown low to moderate levels of confidence in 10-year indicator trend estimates because of low precision [63]. Improving indicator estimate precision would benefit future assessments. Large tracts of BLM-managed lands occur along the boundaries of the 17 parks. As of 2022, there were 3,200 AIMt and LMF observed sites within 40 km of these parks. Filtering BLM sites by ecosite and land-use history to match the range of park conditions would reduce the number of usable sites, but potentially there would be sufficient data to obtain suitable synthetic estimates for at least some of the parks and ecosites for SAE applications.

Incorporating composite estimation into an existing monitoring program can be challenging and requires statistical expertise. There can be many statistical considerations in developing composite estimation applications, such as discerning appropriate methods to correct for under coverage and obtaining MSE weightings when necessary, and selecting between model-assisted (design-based) and model-based (not design-based) methods in SAE applications (e.g., [19]). An extensive technical literature details the statistical issues and options. Identifying opportunities and implementing composite estimation will require the assistance of a statistician familiar with this literature. If compositing complementary information were to become a routine part of a program, ideally developing a repeatable workflow and software tools would ease the burden of acquiring composite estimates by program analysts already saddled with numerous tasks. This is an up-front cost in using composite estimation, but the long-term payback is the consistent acquisition of the best possible estimates for assessing the status and trend of natural resources.

## Supporting information

S1 Appendix Optimal composite weight and precision gain. (DOCX)

S2 Appendix LMF state-level sample weight calculations. (DOCX)

S3 Appendix Method to correct for AIMt under coverage of rangelands. (DOCX)

S1 Code CompositeEstimation.r. R program to derive survey and composite estimates and perform statistical comparisons of indicators between Core and NonCore sagebrush on BLM-managed lands in WAFWA MZ II (2015–18).
(R)

S1 Table AIMt data set. The AIMt analysis data set for Core and NonCore sagebrush on BLM-managed lands in WAFWA MZ II (2015–18) [sheet name—AIMt_WY_MZII].
(XLSX)

S2 Table LMF data set. The LMF analysis data set for Core and NonCore sagebrush on BLM-managed lands in WAFWA MZ II (2015–18) [sheet name—LMF_WY_MZII].
(XLSX)

S3 Table Indicator estimates and Z-score results. Indicator estimates of percent area meeting standards and SEs in Wyoming Core and NonCore on BLM-managed sagebrush communities in WAFWA MZ II (2015–18), and results of the Z-Score test to determine significant differences in indicator estimates between the two conservation areas.
(DOCX)

S1 Fig (ZIP)

## References

[1] Theobald DM, Stevens DL, White D, Urquhart NS, Olsen AR, Norman JB. Using GIS to generate spatially balanced survey designs for natural resource applications. Environ Manage. 2007;40: 134–146. Available from: https://link.springer.com/article/10.1007/s00267-005-0199-x 17546523 10.1007/s00267-005-0199-x

[2] Fancy SG, Gross JE, Carter SL. Monitoring the condition of natural resources in US national parks. Environ Monit Assess. 2009;151: 161–174. Available from: https://link.springer.com/article/10.1007/s10661-008-0257-y 18509737 10.1007/s10661-008-0257-y

[3] Fancy SG, Bennetts RE. Institutionalize an effective long-term monitoring program in the US National Park Service. In: Gitzen RA, Millspaugh JJ, Cooper AB, Licht DS, editors. Design and Analyses of Long-term Ecological Monitoring Studies. UK: Cambridge University Press; 2012. pp. 481–497.

[4] O’Dell T, Garman S, Evenden A, Beer M, Nance E, Perry D, et al. Northern Colorado Plateau Inventory and Monitoring Network, Vital Signs Monitoring Plan. National Park Service, Inventory and Monitoring Network, Moab, UT. National Park Service. 2005. Available from: https://irma.nps.gov/DataStore/Reference/Profile/579097

[5] Kachergis E, Lepak N, Karl M, Miller S, Davidson Z. Guide to Using AIM and LMF Data in Land Health Evaluations and Authorizations of Permitted Uses. Technical Note 453. U.S. Department of the Interior, Bureau of Land Management, National Operations Center, Denver, CO. 2020. Available from: https://www.blm.gov/learn/blm-library/agency-publications/technical-notes

[6] Larsen DP, Olsen AR, Lanigan SH, Moyer C, Jones KK, Kincaid TM. Sound survey designs can facilitate integrating stream monitoring data across multiple programs. J Am Water Resour Assoc. 2007;43: 384–397.

[7] U.S. Agency for HealthCare Research and Quality. Producing state estimates with the Medical Expenditure Panel Survey, Household Component. Medical Expenditure Panel Survey Methodology Report #16. 2005. Available from: https://search.ahrq.gov/search?q=state%20estimates&siteDomain=meps.ahrq.gov

[8] Baldi S (ed). Technical report and data file user’s manual for the 2003 National Assessment of Adult Literacy (NCES 2009–476). U.S. Department of Education, National Center for Education Statistics. Washington, DC: U.S. Government Printing Office. 2009. Available from: https://nces.ed.gov/pubs2009/2009476_1.pdf

[9] Lent J, Miller SM, Cantwell PJ, Duff M. Effects of composite weights on some estimates from the current population survey. J Off Stat. 1999;15: 431–448.

[10] Census Bureau U.S. Current population survey design and methodology, Technical Paper 66, Chapter 10. 2006. Available from: https://www2.census.gov/programs-surveys/cps/methodology/tp-66.pdf

[11] Roesch FA. Composite estimators for growth derived from repeated plot measurements of positively-asymmetric interval lengths. Forests. 2018;9,427. 10.3390/f9070427

[12] Fay RE III, Herriot RA. Estimates of income for small places: an application of James-Stein procedures to census data. J Am Stat Assoc. 1979;74: 269–277. 10.1080/01621459.1979.10482505

[13] Lohr SL, Raghunathan TE. Combining survey data with other data sources. Stat Sci. 2017; 32: 293–312. doi: 10.1214/16-STS584

[14] Rao JNK. Some methods for small area estimation. Riv Int Sci Sociali. 2008;4: 387–406. Available from: https://www.jstor.org/stable/41625216

[15] Moretti A, Whitworth A. Development and evaluation of an optimal composite estimator in spatial microsimulation small area estimation. Geogr Anal. 2020;52: 351–370. 10.1111/gean.12219

[16] Larsen MD. Estimation of small-area proportions using covariates and survey data. J Stat Plan Inference. 2003;112: 89–98. Available from: https://www.sciencedirect.com/science/article/abs/pii/S0378375802003257

[17] Goerndt ME, Monleon VJ, Temesgen H. Small-area estimation of county-level forest attributes using ground data and remote sensed auxiliary information. For Sci. 2013;59: 536–548. 10.5849/forsci.12-073

[18] Cao Q, Dettmann GT, Radtke PJ, Coulston JW, Derwin J, Thomas VA, et al. Increased precision in county-level volume estimates in the United States National Forest Inventory with area-level small area estimation. Front For Glob Change. 2022;5:769917. 10.3389/ffgc.2022.769917

[19] Gt Dettmann, Radtke PJ Coulston JW, Green PC Wilson BT, Moisen GG. Review and synthesis of estimation strategies to meet small area needs in forest inventory. Front For Glob Change. 2022;5:813569. 10.3389/ffgc.2022.813569

[20] Costa A, Satorra A, Ventura E. Improving small area estimation by combining surveys: new perspectives in regional statistics. Sort (Barc). 2006;30: 101–122. 10.2139/ssrn.1002507

[21] MacKinnon WC, Karl JW, Toevs GR, Taylor JJ, Karl M, Spurrier CS, et al. BLM core terrestrial indicators and methods. Technical Note 440. U.S. Department of the Interior, Bureau of Land Management, National Operations Center, Denver, CO. 2011. Available from: https://www.blm.gov/learn/blm-library/agency-publications/technical-notes

[22] Yu CL, Li J, Karl MG, Krueger TJ. Obtaining a balanced area sample for the Bureau of Land Management rangeland survey. J Agric Biol Environ Stat. 2020;25: 250–275. 10.1007/s13253-020-00392-5

[23] Herrick JE, Van Zee JW, McCord SE, Courtright EM, Karl JW, Burkett LM. Monitoring manual for grassland, shrubland, and savanna ecosystems. Second edition, Volume I: Core Methods. USDA- ARC Jornada Experimental Range, Las Cruces, NM. 2017.

[24] State of Wyoming. Greater sage-grouse core area protection. Office of the Governor, Executive Order 2019–3, Cheyenne, Wyoming, USA. 2019. Available from: https://wgfd.wyo.gov/Habitat/Sage-Grouse-Management/Sage-Grouse-Executive-Order

[25] State of Wyoming. Greater sage-grouse core area protection strategy, 2019 conservation and development activities report. Office of the Governor, Executive Order 2019–3, Cheyenne, Wyoming, USA. 2019. Available from: https://wgfd.wyo.gov/Habitat/Sage-Grouse-Management/Sage-Grouse-Executive-Order

[26] Gamo RS, Beck JL. Effectiveness of Wyoming’s sage-grouse core areas: influences on energy development and male lek attendance. Environ Manage. 2017;59: 189–203. doi: 10.1007/s00267-016-0789-9 27826693

[27] Hanser SE, Deibert PA, Tull JC, Carr NB, Aldridge CL, Bargsten TC, et a l. Greater sage-grouse science (2015–17)—Synthesis and potential management implications. U.S. Geological Survey Open-File Report 2018–1017. 2018. Available from: 10.3133/ofr20181017

[28] Smith KT, Beck JL, Pratt AC. Does Wyoming’s core area policy protect winter habitats for Greater Sage-Grouse? Environ Manage. 2016;58: 585–596. doi: 10.1007/s00267-016-0745-8 27515024

[29] Spence ES, Beck JL, Gregory AJ. Probability of lek collapse is lower inside sage-grouse core areas—Effectiveness of conservation policy for a landscape species. PloS ONE. 2017; 12(11): e0185885. doi: 10.1371/journal.pone.0185885 29121066 PMC5679516

[30] Dinkins JB, Smith KT, Beck JL, Kirol CP, Pratt AC, Conover MR. Microhabitat conditions in Wyoming’s sage-grouse core areas: effects on nest site selection and success. PLoS ONE. 2016;11(3): e0150798. doi: 10.1371/journal.pone.0150798 27002531 PMC4803343

[31] Stiver S, Apa A, Bohne J, Bunnell S, Deibert P, Gardner S, et al. Greater sage-grouse comprehensive conservation strategy. Western Association of Fish and Wildlife Agencies, Unpublished Report, Cheyenne, Wyoming, USA. 2006. Available from: https://wdfw.wa.gov/publications/01317

[32] Carr NB, Sherrill KR, George TL, Garman SL. Sagebrush steppe. In: Carr NB and Melcher CP, editors. Wyoming Basin Rapid Ecoregional Assessment: (ver. 1.1, April 2017). U.S. Geological Survey Open-File Report 2015–1155; 2017. pp. 287–314. 10.3133/ofr20151155

[33] Gilbert MM, Chalfoun AD. Energy development affects populations of sagebrush songbirds in Wyoming. J Wildl Manage. 2011;75: 816–824. 10.1002/jwmg.123

[34] Germaine SS, Assal T, Freeman A, Carter SK. Distance effects of gas field infrastructure on pygmy rabbits in southwestern Wyoming. Ecosphere. 2020;11(8):e03230. doi: 10.1002/ecs2.3230

[35] LANDFIRE. Conterminous U.S. LANDFIRE LF 2016 Remap (LF_200) Vegetation us_200 Existing Vegetation Type. Available from: https://landfire.gov/lf_remap.php

[36] Knight DH, Jones GP, Reiners WA, Romme WH. Mountains and plains: the ecology of Wyoming landscapes. 2nd ed. New Haven: Yale University Press; 2014.

[37] Grafström A, Ekström M, Jonsson BG, Esseen P-A, Ståhl G. On combining independent probability samples. Surv Methodol. 2019;45: 349–364. Available from: https://www150.statcan.gc.ca/n1/en/pub/12-001-x/2019002/article/00003-eng.pdf?st=1RIvUXCi

[38] Graybill FA, Deal RB. Combining unbiased estimators. Biometrics. 1959;15: 543–550. 10.2307/2527652

[39] Kuo L. Composite estimation of totals for livestock surveys. J Am Stat Assoc. 1989;84: 421–429. 10.1080/01621459.1989.10478786

[40] Stevens DL Jr, Olsen AR. Variance estimation for spatially balanced samples of environmental resources. Environmetrics. 2003;14: 593–610. 10.1002/env.606

[41] Kott PS. The delete-a-group jackknife. J Off Stat. 2001;17: 521–526. Available from: https://www.scb.se/contentassets/ca21efb41fee47d293bbee5bf7be7fb3/the-delete-a-group-jackknife.pdf

[42] Taylor JJ, Kachergis EJ, Toevs GR, Karl JW, Bobo MR, Karl M, et al. AIM-monitoring: A component of the BLM Assessment, Inventory, and Monitoring strategy. Technical Note 445. U.S. Department of the Interior, Bureau of Land Management, National Operations Center, Denver, CO. 2014. Available from: https://www.blm.gov/learn/blm-library/agency-publications/technical-notes

[43] Toevs GR, Karl JW, Taylor JJ, Spurrier CS, Karl M, Bobo MR, et al. Consistent indicators and methods and a scalable sample design to meet assessment, inventory, and monitoring information needs across scales. Rangelands. 2011;33: 14–20. 10.2111/1551-501X-33.4.14

[44] Toevs GR, Taylor JJ, Spurrier CS, MacKinnon WC, Bobo MR. Bureau of Land Management Assessment, Inventory, and Monitoring strategy: For integrated renewable resources management. Bureau of Land Management, National Operations Center, Denver, CO. 2011. Available from: https://www.blm.gov/sites/blm.gov/files/uploads/IB2012-080_att1.pdf

[45] Stevens DL, Olsen AR. Spatially balanced sampling of natural resources. J Am Stat Assoc. 2004;99: 262–278. Available from: https://www.jstor.org/stable/27590371

[46] Natural Resources Conservation Service. National Resources Inventory Grazing Land On-Site Data Collection Handbook of Instructions. U.S. Dept. of Agriculture, Natural Resources Conservation Service. 2024. Available from: https://grazingland.cssm.iastate.edu/files/inline-files/NRIGrazingLandHandbook2024_0.pdf

[47] Goertel M, Fahrer B, Marzluf J, Oldenburg R. Appendix 14: Wyoming State Office Monitoring Report for the 2015 Wyoming Land Use Plan Amendments and Revisions 2015–2020. In: Herren V, Kachergis E, Titolo A, Mayne K, Glazer K, Lambert K, et al., editors. Greater sage-grouse plan implementation: Rangewide monitoring report for 2015–2021. U.S. Department of the Interior, Bureau of Land Management, Denver, CO; 2021. pp. 1–24. Available from: https://eplanning.blm.gov/public_projects/2016719/200502020/20050224/250056407/Greater%20Sage-Grouse%20Five-year%20Monitoring%20Report%202020.pdf

[48] Bureau of Land Management. Standards for healthy rangelands and guidelines for livestock grazing management for public lands administered by the Bureau of Land Management in the State of Wyoming. 1997. Available from: https://www.blm.gov/sites/blm.gov/files/documents/files/PublicRoom_Wyoming_StandardsandGuidelinesforHealthyRangelands1997.pdf

[49] Dumelle M, Kincaid TM, Olsen AR, Weber MH. spsurvey: Spatial Sampling Design and Analysis. R package version 5.3.0. 2022. 10.18637/jss.v105.i03 PMC9926341 36798141

[50] R Core Team. R: A language and environment for statistical computing. R Foundation for Statistical Computing, Vienna, Austria. 2022.

[51] Urquhart SN. The role of monitoring design in detecting trend in long-term ecological monitoring studies. In: Gitzen RA, Millspaugh JJ, Cooper AB, Licht DS, editors. Design and Analyses of Long-term Ecological Monitoring Studies. UK: Cambridge University Press; 2012. pp. 151–173.

[52] Garman SL, Schweiger EW, Manier DJ. Simulating future uncertainty to guide the selection of survey designs for long-term monitoring. In: Gitzen RA, Millspaugh JJ, Cooper AB, Licht DS, editors. Design and Analyses of Long-term Ecological Monitoring Studies. UK: Cambridge University Press; 2012. pp. 228–249.

[53] Bowker MA, Miller ME, Belote TR, Garman SL. Ecological thresholds as a basis for defining management triggers for National Park Service vital signs–Case studies for dryland systems. U.S. Geological Survey Open-File Report 2013–1244. 2013. Available from: https://pubs.usgs.gov/of/2013/1244/

[54] Kershner JL, Archer EK, Coles‐Ritchie M, Cowley ER, Henderson RC, Kratz K, et al. Guide to effective monitoring of aquatic and riparian resources. Gen Tech Rep RMRS-GTR-121. Fort Collins, CO. U.S. Department of Agriculture, Forest Service, Rocky Mountain Research Station. 2004. 10.2737/RMRS-GTR-121

[55] Isaak DJ, Young MK, McConnell C, Roper BB, Archer EK, Staab B, et al. Crowd-sourced databases as essential elements for Forest Service partnerships and aquatic resource conservation. Fisheries. 2018;43: 424–430. 10.1002/fsh.10083

[56] Reeves GH, Hohler DB, Larsen DP, Busch DE, Kratz K, Reynolds K, et al. Effectiveness monitoring the aquatic and riparian component of the Northwest Forest Plan: conceptual framework and options. Gen Tech Rep PNW-GTR-577. Portland, OR. U.S. Department of Agriculture, Forest Service, Pacific Northwest Research Station. 2004. Available from: https://www.fs.usda.gov/pnw/pubs/pnw_gtr577.pdf

[57] Bureau of Land Management. AIM National Aquatic Monitoring Framework: Field protocol for wadeable lotic systems. Technical Reference 1735–2, version 2. U.S. Department of the Interior, Bureau of Land Management, National Operations Center, Denver, CO. 2021. Available from: https://www.blm.gov/learn/blm-library/agency-publications/technical-references

[58] Scully RA, Dlabola EK, Heaston ED, Bayer JM, Courtwright JL, Snyder MN, et al. Wadeable stream habitat data integrated from multiple monitoring programs for the US from 2000–2022. U.S. Geological Survey data release. 2023. doi: 10.5066/P9J3P7SN

[59] Bayer JM, Scully RA, Dlabola EK, Courtwright JL, Hirsch CL, Hockman-Wert D, et al. Sharing FAIR monitoring program data improves discoverability and reuse. Environ Monit Assess. 2023;195:1141. doi: 10.1007/s10661-023-11788-4 37665400

[60] Acker SA, Boetsch JR, Fallon B, Denn M. Stable background tree mortality in mature and old-growth forests in western Washington (NW USA). For Ecol Manage. 2023;532 (2023) 120817. doi: 10.1016/j.foreco.2023.120817

[61] Witwicki D, Thomas H, Weissinger R, Wight A, Topp S, Garman SL, et al. Upland vegetation and soils monitoring protocol for park units in the Northern Colorado Plateau Network: Version 1.07. Natural Resource Report NPS/NCPN/NRR—2017/1570. National Park Service, Fort Collins, Colorado. 2017. Available from: https://www.nps.gov/im/reports-nrr.htm

[62] DeCoster JK, Lauver CL, Miller ME, Norris JR, Snyder AEC, Swan MC, et al. Integrated upland monitoring protocol for the Southern Colorado Plateau Network. Natural Resource Report NPS/SCPN/NRR–2012/577. National Park Service, Fort Collins, Colorado. 2012. Available from: https://www.nps.gov/im/reports-nrr.htm

[63] Witwicki DL. Status and trends in upland vegetation and soils at Capitol Reef National Park, 2009–2018 (revised with cost estimate). Natural Resource Report NPS/NCPN/NRR—2020/2183. National Park Service, Fort Collins, Colorado. 2020. doi: 10.36967/nrr-2279505

