# Supplementary material for: Composite estimation to combine spatially overlapping environmental monitoring surveys
Source: PLoS One. 2024 Mar 22;19(3):e0299306. doi: 10.1371/journal.pone.0299306 (PMC10959383; doi:10.1371/journal.pone.0299306)
Supplement: S1 Appendix — (DOCX) [file pone.0299306.s001.docx]

**S1 Appendix. Optimal composite weight and precision gain.**

**I. Optimal composite weight and standard error**

The narrative equations for variance of a composite estimate and the composite weight $\lambda$ are repeated to facilitate understanding the demonstration of optimal weightings. The variance of a composite estimate is:

$\hat{V}\left( Ŷ_{c} \right){=\lambda}^{2} \hat{\sigma}_{Ŷ_{1}}^{2}+\left( 1-\lambda\right)^{2} \hat{\sigma}_{Ŷ_{2}}^{2},$ (A1) where $\hat{\sigma}_{Ŷ_{1}}$is the standard error (SE) of the survey #1 estimate and $\hat{\sigma}_{Ŷ_{2}}$is the SE of the survey #2 estimate. The SE of a composite estimate is $\hat{\sigma}_{c}=\sqrt{\hat{V}\left( Ŷ_{c} \right)}$. A variance-weighted λ that maximizes precision (minimize variance) is obtained by:

$\lambda=\frac{\hat{\sigma}_{Ŷ_{2}}^{2}}{\hat{\sigma}_{Ŷ_{1}}^{2}+\hat{\sigma}_{Ŷ_{2}}^{2}}$ . (A2)

The minimal variance of the composite estimate can be rewritten as $\hat{V}\left( \hat{Y}_{c} \right)=\frac{\hat{\sigma}_{\hat{Y}_{1}}^{2}\hat{\sigma}_{Ŷ_{2}}^{2}}{\hat{\sigma}_{Ŷ_{1}}^{2}+\hat{\sigma}_{Ŷ_{2}}^{2}}$. By rearranging this formula as $\hat{\sigma}_{Ŷ_{1}}^{2}\times\frac{\hat{\sigma}_{Ŷ_{2}}^{2}}{\hat{\sigma}_{Ŷ_{1}}^{2}+\hat{\sigma}_{Ŷ_{2}}^{2}}$ or $\hat{\sigma}_{Ŷ_{2}}^{2}\times\frac{\hat{\sigma}_{Ŷ_{1}}^{2}}{\hat{\sigma}_{Ŷ_{1}}^{2}+\hat{\sigma}_{Ŷ_{2}}^{2}}$, it becomes readily apparent that $\hat{V}\left( Ŷ_{c} \right)$ is always smaller than either $\hat{\sigma}_{Ŷ_{1}}^{2}$ or $\hat{\sigma}_{Ŷ_{2}}^{2}$since the ratio multiplier is smaller than one.

The variance (squared standard error) of a composite estimate can be also written as,

$\hat{V}\left( Ŷ_{c} \right)=\left( \hat{\sigma}_{Ŷ_{1}}^{2}+\hat{\sigma}_{Ŷ_{1}}^{2} \right)\lambda^{2}-2 \hat{\sigma}_{Ŷ_{2}}^{2}\lambda+\hat{\sigma}_{Ŷ_{2}}^{2},$ (A3)

which is a parabola with its lowest point (optimal λ) between 0 and 1. The end points of 0 and 1 are two theoretical cases where only one of the two surveys is used in composite estimation. The relationship between λ and the variance of a composite estimate can be shown by using alternative values of λ for a fixed ratio of the two survey estimate variances (Fig S1A). At the left end of the curve (λ=0), the composite variance is the same as $\hat{\sigma}_{Ŷ_{2}}^{2}$. Then it decreases to a minimum as λ reaches the optimal value (Eq (A2)). The composite variance increases to $\hat{\sigma}_{Ŷ_{1}}^{2}$ at the right end of the curve (λ=1). If variances of the survey estimates are identical (Fig S1A, line(b)), the best λ is 0.5 which achieves a balanced weighting of the two survey estimate variances. The optimal λ will be closer to 0 if $\hat{\sigma}_{Ŷ_{1}}^{2}$ is the smaller variance (Fig S1A, line(a)), and closer to 1 if $\hat{\sigma}_{Ŷ_{2}}^{2}$ is the smaller variance (Fig S1A, line(c)).


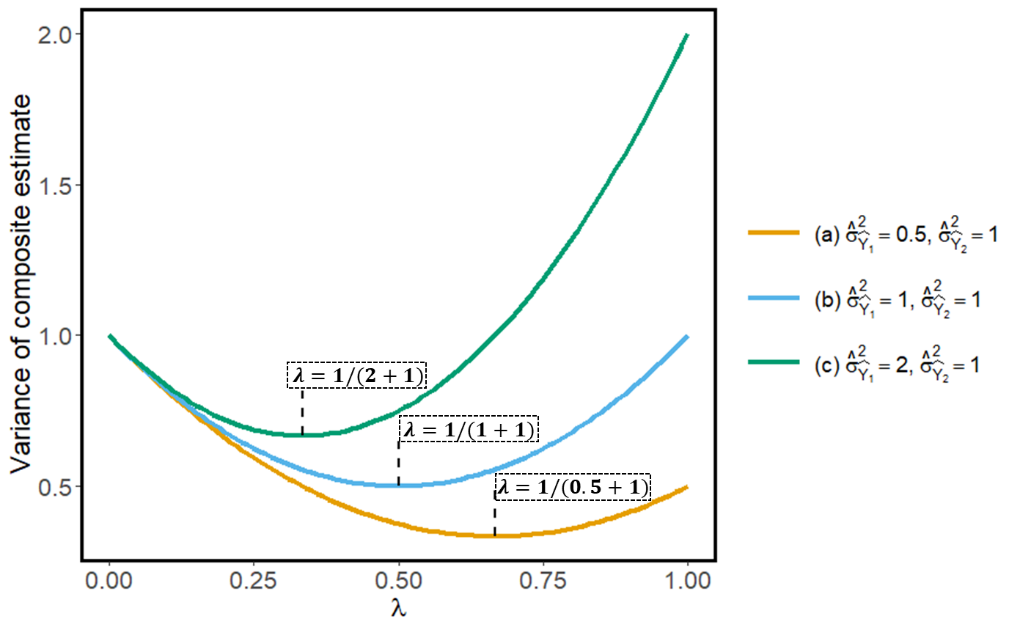


**Fig S1A. Changes in the variance of a composite estimate over different values of λ.** The variance of survey #2 ($\hat{\sigma}_{Ŷ_{2}}^{2}$) was set to one and the variance of survey #1 ($\hat{\sigma}_{Ŷ_{1}}^{2}$) was (a) 0.5, (b) 1.0, and (c) 2.0. The optimal λ for each combination of variances is the bottom of the dashed line below the Eq (A2) solution.

**II. Precision gain**

Precisions are known as the reciprocal of variances with larger values preferable. We can calculate the precision gain by taking the ratio of precisions. For example, the most balanced situation is when variances of the two surveys are equal ($\hat{\sigma}_{Ŷ_{1}}^{2}=\hat{\sigma}_{Ŷ_{2}}^{2}$). In this case, the relative precision of a composite estimate relative to each of the survey is 200% ($\frac{1}{\hat{\sigma}_{c}^{2}}/\frac{1}{\hat{\sigma}_{Ŷ_{1}}^{2}}=\frac{1}{\hat{\sigma}_{c}^{2}}/\frac{1}{\hat{\sigma}_{Ŷ_{2}}^{2}}=2$), which equates to a precision gain of 100%.

The ratio $\hat{\sigma}_{Ŷ_{1}}^{2}/\hat{\sigma}_{Ŷ_{2}}^{2}$ determines how much precision gain can be obtained with composite estimation. The precision gain of a composite estimate relative to the precision of the two survey estimates over different values of the ratio is shown in Fig S1B. When survey #1 is more precise ($\hat{\sigma}_{Ŷ_{1}}^{2}/\hat{\sigma}_{Ŷ_{2}}^{2}$ is smaller than 1), the composite estimate has less precision gain relative to survey #1 (Fig S1B, grey solid line) and more precision gain relative to survey #2 (Fig S1B, blue dotted line). An example of such a case is also provided in Fig S1A, line (a). When survey #2 is more precise ($\hat{\sigma}_{Ŷ_{1}}^{2}/\hat{\sigma}_{Ŷ_{2}}^{2}$ is greater than 1), the composite estimate has higher precision gain relative to survey #1 (Fig S1B, grey dashed line) and less precision gain relative to survey #2 (Fig S1B, blue solid line). Theoretically, as the ratio $\hat{\sigma}_{Ŷ_{1}}^{2}/\hat{\sigma}_{Ŷ_{2}}^{2}$ deviates from the most balanced situation ($\hat{\sigma}_{Ŷ_{1}}^{2}/\hat{\sigma}_{Ŷ_{2}}^{2}=1$), the composite precision gain relative to the more accurate survey (solid segments) diminishes, while precision gain relative to the less accurate survey (dashed segments) increases.


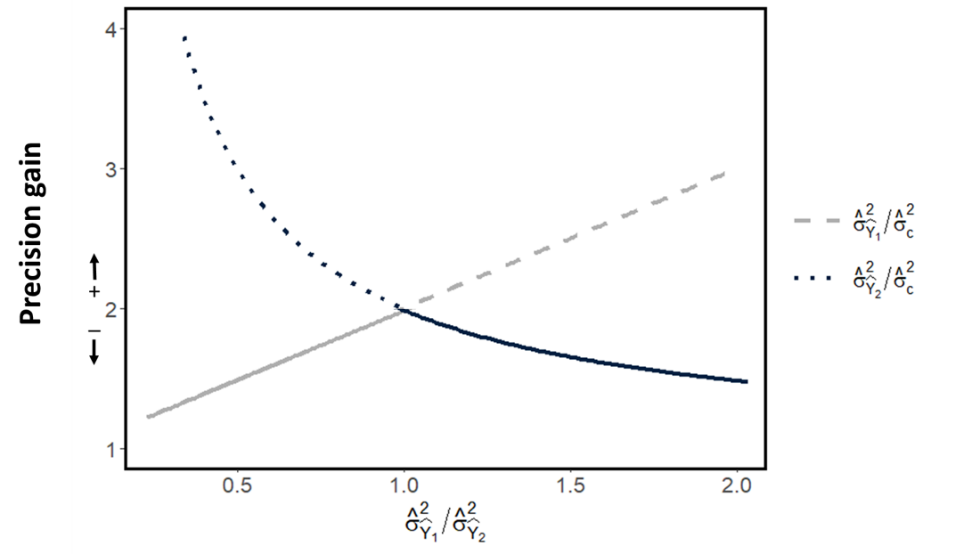


**Fig S1B. Precision gain of a composite estimate relative to the precision of two survey estimates.** The $\hat{\sigma}_{Ŷ_{1}}^{2}/\hat{\sigma}_{Ŷ_{2}}^{2}$ is the ratio between precision of survey #2 and precision of survey #1. The solid lines identify the more precise survey.
