# Supplementary material for: Composite estimation to combine spatially overlapping environmental monitoring surveys
Source: PLoS One. 2024 Mar 22;19(3):e0299306. doi: 10.1371/journal.pone.0299306 (PMC10959383; doi:10.1371/journal.pone.0299306)
Supplement: S2 Appendix — (DOCX) [file pone.0299306.s002.docx]

**S2 Appendix. LMF state-level sample weight calculations.**

**I. LMF survey design background**

The LMF program uses state-specific 10-year (2014-2023) surveys but design features are consistent among states. Surveys are two-stage cluster designs. A survey starts with a land frame consisting of all BLM-managed lands within a state and divides a frame into the following:

- Strata. Type I (primary sage-grouse habitat) and Type II (other) strata.
- Thiessen polygons (TPs). These are a device for spatial stratification of the sample sites. About equal-sized TPs are constructed within a strata with more and smaller TPs in Type I than in Type II strata to increase the density of sites in prime sage-grouse habitat.
- Segments. For each TP, 20 ~65-ha rectangular segments (Public Land Survey System quarter-sections) are randomly selected for the 10-year survey (see Yu et al. [22] for details of selection procedures).

Three sites are randomly located within each segment [22]. Two sites are to be observed. The third site is an oversample and observed if one or both of the first two sites fail to be observed. Response status of sites is recorded in the field during the annual monitoring event to indicate if a site is rangeland on BLM-managed lands, not rangeland but on BLM-managed lands, and not on BLM-managed land (frame error). Additionally, response status indicates if a site was observed or unobserved. All allocated annual sample sites on BLM-managed land are scored as rangeland or other even if they were not observed. This provides a comprehensive estimate of rangeland area.

Calculating LMF sample weights requires the size of TPs and segments, and response codes of every site in an allocated annual monitoring event. All of this information is available in a state 10-year Master Sample and associated databases.

**II. Sample weight calculations**

The LMF program provides scalable sample weights for a state, ecoregions, and sage-grouse management zones [22] using complex multi-scale calibrations. The state-level procedures described here are a stream-lined version of LMF’s procedures for only sample weights within a state and does not involve multi-scale adjustments. Sample weights are derived for a specific application using the data from one or more annual monitoring event(s). Applications may use all BLM-managed land in a state or a subset as the analysis area. Calculations use the response codes of all sites and the GIS areal measures of BLM-managed land in the analysis area. Final sample weights are generated only for observed sites. These weights are used in statistical analyses of the observations.

Calculations follow equations 7, 8, 9, and 15 in Yu et al. [22] with modifications to simplify obtaining state-level sample weights and weights for post-stratified domains when these are used in an application.

**1**. Initial site weights are derived from the LMF state design and are invariant of the application area and any post-stratified analysis domains. Initial weights are derived from the sampling probabilities of the LMF design. Define $h\left( j \right)$ as the TP containing segment *j*. The selection probability, $\pi,$of segment j is derived as;

$\pi_{j}=\frac{\sum_{l\epsilon M_{h(j)}} A_{l}}{A_{h(j)}}$ X $\frac{2Y}{20}$ , (A1)

where $A_{l}$ is area of segment *l*, $A_{h(j)}$ is the area of $h(j)$, M*_h(j)_* is the set of all segments in $h(j)$ in the Master Sample, and $Y$ is the time interval (no. of yrs) of the analysis data set. The first ratio in Eq (A1) is the probability of selecting the 10-year Master Sample. The second ratio is the probability of selecting samples out of the Master Sample, where 2Y is the standard annual allocation of 2 segments times the number of annual surveys (Y) in an analysis, and 20 is the total number of segments per TP.

The initial weight for site k in segment j is;

$w_{jk}=$ $\frac{1}{\pi_{j}}$X $\frac{A_{j}}{3}$, (A2)

where A_j_ is the area (ha) of segment j and k is site 1, 2, and 3.

Subsequent calculations are specific to the application area and only use the portion of the state-wide sample and of the state-wide frame within this area. Calculations are applied separately to each LMF stratum. To simplify notation, the following equations do not include strata identifiers. Therefore, it is implicit that calculations only use the segments, sites, and spatial extent of BLM-managed lands that overlap a stratum. For analyses with post-stratified domains, the following calculations are repeated for each of the domain-LMF stratum combinations, where similarly, calculations only use the segments, sites, and extent of BLM-managed lands that overlap a domain-LMF stratum combination.

**2**. The initial weights are ratio-adjusted using BLM-managed land area as a control total by;

$w_{jk}^{T}=T \times\left\{ \sum_{\left( jk \right)\epsilon S} \left( \delta_{1, jk}+ \delta_{2, jk}+ \delta_{3, jk} \right)w_{jk} \right\}^{-1}\times$ $w_{jk}$, (A3)

where $w_{jk}^{T}$is the BLM land weight (ha) for site k in segment j, $T$ is the BLM land area (ha), S is the set of all sites, and $\delta_{1-3}$are response indicators, where $\delta_{1}$is 1 if a site is observed rangeland else 0, $\delta_{2}$ is 1 if a site is unobserved rangeland else 0, and $\delta_{3}$ is 1 if a site is not rangeland but on BLM-managed lands else 0. This adjustment eliminates sites not on BLM-managed lands. The sum of BLM land weights equals the total extent of BLM-managed lands (T).

**3**. A similar weight adjustment to rangeland area is required, but a state frame does not explicitly map rangelands. Rangeland area (ha), $\hat{T}_{r}$, must be estimated from the sample of an application by;

$\hat{T}_{r}=\sum_{\left( jk \right)\epsilon S} \left( \delta_{1, jk}+ \delta_{2, jk} \right)w_{jk}^{T}$. (A4)

**4**. Using the rangeland area estimate, the BLM land weights are ratio-adjusted to rangeland area by;

$w_{jk}^{R}=\hat{T}_{r} \times\left\{ \sum_{\left( jk \right)\epsilon S} \delta_{1, jk} w_{jk}^{T} \right\}^{-1}{\times w}_{jk}^{T}$ , (A5)

where $w_{jk}^{R}$is the final rangeland weight (ha) of site k in segment j. The sum of the rangeland weights across strata is the estimated rangeland area (ha). When using post-stratified analysis domains, the sum of rangeland weights across LMF strata within a domain is the estimated rangeland area in the domain.

**III. Replicate sample weights**

Replicate sample weights are based on the segments assigned to a replicate. Because a replicate is a subset of segments in a sample, the number of allocated segments in some TPs will be fewer than 2Y (Eq (A1)). That is, the segments excluded in a replicate are treated as if they were not allocated. All other design attributes do not change, such as the total number of segments per TP in the Master Sample. To calculate replicate weights, the initial selection probability of segment j is derived by;

$\pi_{j}=\frac{\sum_{l\epsilon M_{h(j)}} A_{l}}{A_{h(j)}}$ $\times$ $\frac{{Ns}_{h(j)}}{20}$ , (A6)

where ${Ns}_{h(j)}$ is the number of allocated segments in a replicate in the TP containing segment j. Equations (A2-A5) are then used to derive replicate weights.
