# Supplementary material for: Composite estimation to combine spatially overlapping environmental monitoring surveys
Source: PLoS One. 2024 Mar 22;19(3):e0299306. doi: 10.1371/journal.pone.0299306 (PMC10959383; doi:10.1371/journal.pone.0299306)
Supplement: S3 Appendix — (DOCX) [file pone.0299306.s003.docx]

**S3 Appendix. Method to correct for AIMt under coverage of rangelands.**

**I. Background**

Survey design and protocol differences can result in AIMt areal estimates of rangelands lower than those of LMF. This is referred to as under coverage. The sources of AIMt under coverage are inaccessible sites and strata without any sites in a clipped analysis area even though unclipped strata had sites. A method was developed to correct for AIMt under coverage in the case study.

The AIMt survey used in the study was an aggregate of eight independent, non-overlapping BLM field office surveys (samples and frames). Calibration of AIMt weights must be done using site and strata information for the source field office survey. Each site and strata in the aggregate AIMt survey were codified to identify sites and strata of each field office survey for this purpose.

Response status of AIMt sites were observed rangeland, non-target which included observed non-rangeland sites and sites that were not upland vegetation, and inaccessible (IA) which lacked observations and information about the vegetation community of a site.

**II. Under coverage correction procedures**

Our method assumed that sources of under coverage, the IA sites and strata with no sites, were largely rangelands. This is a reasonable assumption given the extent of rangelands in the study area. We refer to the collective sample weights of IA sites and area of strata with no sites as unsampled area. Procedures used the AIMt Core sample and frame and the AIMt NonCore sample and frame. The terms sample and frame, hereafter, refer to these. We refer to Core and NonCore as domains. The sites of a sample and strata of a frame in the same field office (FO) are called a FO subset. Our correction method allocated under coverage area among the eight FO subsets of a sample proportional to the amount of unsampled area in a FO subset, such that subsets with larger amounts were allocated a larger portion of under coverage than those with smaller amounts. The added rangeland area was used to calibrate the sample weights of observed sites which corrected for under coverage.

The known under coverage area (ha) in domain *d*, ${UC}_{d}$, was obtained as the difference between LMF and the initial AIMt rangeland area estimates. We determined unsampled area for each FO subset by domain, ${US}_{df}$ where $f$is FO subset 1, 2,..8, and obtained the total amount of unsampled area in a domain, ${TUA}_{d}$. The ratio of ${US}_{df}$ and ${TUA}_{d}$ was the relative proportion of unsampled area in FO subset $f$in domain *d*, and was used to allocate known under coverage area by,

${RA}_{df}={UC}_{d} \frac{{US}_{df}}{{TUA}_{d}}$ , (A1)

where ${RA}_{df}$is the rangeland under coverage area in domain *d* that is allocated (i.e., added) to subset $f$ in domain *d*. The addition of ${RA}_{df}$ to the weight sum of observed sites in FO subset $f$ in domain *d* provides an adjusted estimate of rangeland area which is used as a control total for ratio-adjusting the sample weights of observed sites in a FO subset, providing adjusted rangeland weights. The rangeland area estimates of a sample (sum of all adjusted rangeland weights) then equals LMF’s estimates. Our proportional allocation method ensured that the sum of adjusted rangeland weights plus the weights of any non-target sites in a FO subset never exceeded the subset’s total frame area.
