# Supplementary material for: Composite estimation to combine spatially overlapping environmental monitoring surveys
Source: PLoS One. 2024 Mar 22;19(3):e0299306. doi: 10.1371/journal.pone.0299306 (PMC10959383; doi:10.1371/journal.pone.0299306)
Supplement: S3 Table — Indicator estimates of percent area meeting standards and SEs in Wyoming Core and NonCore on BLM-managed sagebrush communities in WAFWA MZ II (2015–18), and results of the Z-Score test to determine significant differences in indicator estimates between the two conservation areas. (DOCX) [file pone.0299306.s007.docx]

**S7 Table**. **Indicator** **Estimates and Z-Score test results.** Indicator estimates of percent area meeting standards and SEs in Wyoming Core and NonCore sagebrush communities on BLM-managed lands in WAFWA MZ II (2015-18), and results of the Z-Score test to determine differences in indicator estimates between the two conservation areas. Estimates and Z-Scores and their significance are shown for composite estimates (**S7 Table A**), for the AIMt survey (**S7 Table B**), and for the LMF survey (**S7 Table C**). Total sample sizes for composite estimates were 1000 in Core and 624 in NonCore, for AIMt survey estimates were 539 in Core and 249 in NonCore, and for LMF survey estimates were 461 in Core and 375 in NonCore. N is the number of sites meeting standards for each indicator. The difference between total sample size and N is the number of sites not meeting indicator standards. The *p*-value of the Z-Score is for a 2-tailed test of significance.

**S7 Table A. Composite estimates.**

| Indicator (units) | Core | | | NonCore | | | Z-score | *p*-value |
| --- | --- | --- | --- | --- | --- | --- | --- | --- |
|  | N | Estimate | SE | N | Estimate | SE |  |  |
| Total foliar cover (%) | 916 | 90.20 | 0.98 | 507 | 76.97 | 2.45 | 5.008 | <0.001 |
| Perennial grass cover (%) | 959 | 95.11 | 0.75 | 520 | 80.28 | 2.00 | 6.952 | <0.001 |
| Sagebrush cover (%) | 687 | 64.79 | 1.99 | 367 | 56.64 | 3.22 | 2.154 | 0.031 |
| Shrub cover (%) | 498 | 53.59 | 2.17 | 286 | 46.26 | 2.99 | 1.983 | 0.047 |
| Perennial forb cover (%) | 352 | 38.17 | 2.14 | 190 | 24.89 | 2.26 | 4.266 | <0.001 |
| Annual forb-grass cover (%) | 265 | 29.39 | 2.05 | 199 | 35.80 | 3.10 | 1.728 | 0.084 |
| Large foliar gaps cover (%) | 937 | 93.35 | 0.85 | 503 | 79.14 | 1.74 | 7.337 | <0.001 |
| Bare ground cover (%) | 974 | 97.11 | 0.60 | 579 | 91.16 | 1.67 | 3.354 | <0.001 |
| Sagebrush shrub height (cm) | 916 | 91.46 | 1.17 | 525 | 83.90 | 2.44 | 2.795 | 0.005 |
| Shrub height (cm) | 180 | 21.16 | 1.94 | 177 | 32.69 | 2.66 | 3.495 | <0.001 |
| Perennial grass height (cm) | 936 | 93.54 | 0.88 | 529 | 88.28 | 1.72 | 2.717 | 0.007 |
| Herbaceous height (cm) | 913 | 90.53 | 1.21 | 510 | 82.89 | 2.10 | 3.152 | 0.002 |
| Soil aggregate stability (index) | 539 | 50.46 | 2.14 | 328 | 52.11 | 3.13 | 0.436 | 0.662 |

**S7 Table B. AIMt survey estimates.**

| Indicator (units) | Core | | | NonCore | | | Z-score | *p*-value |
| --- | --- | --- | --- | --- | --- | --- | --- | --- |
|  | N | Estimate | SE | N | Estimate | SE |  |  |
| Total foliar cover (%) | 509 | 91.62 | 1.65 | 221 | 76.99 | 4.97 | 2.791 | 0.005 |
| Perennial grass cover (%) | 525 | 95.74 | 1.25 | 223 | 75.99 | 5.14 | 3.734 | <0.001 |
| Sagebrush cover (%) | 370 | 65.24 | 2.52 | 149 | 57.48 | 5.10 | 1.366 | 0.172 |
| Shrub cover (%) | 294 | 56.54 | 2.59 | 124 | 48.68 | 5.15 | 1.365 | 0.172 |
| Perennial forb cover (%) | 225 | 41.96 | 2.59 | 98 | 35.32 | 4.81 | 1.216 | 0.224 |
| Annual forb-grass cover (%) | 146 | 29.95 | 2.45 | 88 | 44.17 | 5.26 | 2.450 | 0.014 |
| Large foliar gaps cover (%) | 509 | 92.69 | 1.48 | 209 | 71.47 | 5.22 | 3.911 | <0.001 |
| Bare ground cover (%) | 531 | 97.67 | 0.86 | 237 | 87.98 | 4.00 | 2.369 | 0.018 |
| Sagebrush shrub height (cm) | 487 | 90.54 | 1.53 | 209 | 84.45 | 3.79 | 1.490 | 0.136 |
| Shrub height (cm) | 83 | 19.98 | 2.24 | 66 | 32.02 | 5.09 | 2.163 | 0.031 |
| Perennial grass height (cm) | 507 | 93.25 | 1.30 | 230 | 93.33 | 2.27 | 0.028 | 0.978 |
| Herbaceous height (cm) | 497 | 90.75 | 1.55 | 218 | 87.71 | 3.29 | 0.839 | 0.402 |
| Soil aggregate stability (index) | 291 | 49.84 | 2.62 | 126 | 48.60 | 5.16 | 0.214 | 0.830 |

**S7 Table C. LMF survey estimates.**

| Indicator (units) | Core | | | NonCore | | | Z-score | *p*-value |
| --- | --- | --- | --- | --- | --- | --- | --- | --- |
|  | N | Estimate | SE | N | Estimate | SE |  |  |
| Total foliar cover (%) | 407 | 89.42 | 1.22 | 286 | 76.96 | 2.82 | 4.058 | <0.001 |
| Perennial grass cover (%) | 434 | 94.77 | 0.93 | 297 | 81.05 | 2.17 | 5.813 | <0.001 |
| Sagebrush cover (%) | 317 | 64.04 | 3.23 | 218 | 56.09 | 4.15 | 1.513 | 0.130 |
| Shrub cover (%) | 204 | 46.64 | 3.97 | 162 | 45.03 | 3.68 | 0.298 | 0.766 |
| Perennial forb cover (%) | 127 | 29.97 | 3.81 | 92 | 21.93 | 2.56 | 1.751 | 0.080 |
| Annual forb-grass cover (%) | 119 | 28.10 | 3.71 | 111 | 31.36 | 3.83 | 0.612 | 0.540 |
| Large foliar gaps cover (%) | 428 | 93.67 | 1.04 | 294 | 80.10 | 1.85 | 6.410 | <0.001 |
| Bare ground cover (%) | 443 | 96.57 | 0.84 | 342 | 91.83 | 1.84 | 2.347 | 0.019 |
| Sagebrush shrub height (cm) | 429 | 92.73 | 1.79 | 316 | 83.52 | 3.19 | 2.517 | 0.012 |
| Shrub height (cm) | 97 | 24.72 | 3.89 | 111 | 32.94 | 3.13 | 1.649 | 0.099 |
| Perennial grass height (cm) | 429 | 93.78 | 1.19 | 299 | 81.40 | 2.65 | 4.259 | <0.001 |
| Herbaceous height (cm) | 416 | 90.18 | 1.96 | 292 | 79.57 | 2.73 | 3.157 | 0.002 |
| Soil aggregate stability (index) | 248 | 51.72 | 3.73 | 202 | 54.17 | 3.94 | 0.450 | 0.652 |
